# Association of RSV-related hospitalization and non-compliance with Palivizumab among commercially insured infants: a retrospective claims analysis

**DOI:** 10.1186/1471-2334-13-334

**Published:** 2013-07-19

**Authors:** Dan L Stewart, Kellie J Ryan, Jerry G Seare, Brett Pinsky, Laura Becker, Michael Frogel

**Affiliations:** 1 Department of Pediatrics, University of Louisville School of Medicine, 571 S. Floyd Street, Louisville, KY 40202, USA; 2 Kosair Children’s Hospital, Louisville, KY, USA; 3 Health Outcomes and Pharmacoeconomics, MedImmune, Gaithersburg, MD, USA; 4 Optum, Eden Prairie, MN, USA; 5 Division of General Pediatrics, Steven and Alexandra Cohen Children’s Medical Center, New Hyde Park, NY, USA

**Keywords:** Respiratory syncytial viruses, Palivizumab, Patient compliance, Hospitalization, Infants

## Abstract

**Background:**

Palivizumab has been shown to decrease the incidence of hospitalization due to respiratory syncytial virus (RSV) in infants at risk of severe RSV disease. We examined the association between compliance with palivizumab dosing throughout the RSV season and risk of RSV-related hospitalization in clinical practice.

**Methods:**

Subjects who were born and discharged from the hospital before the RSV season and received ≥1 palivizumab dose during their first RSV season were identified from a large US commercial health insurance database between 01/01/03 and 12/31/09. Subjects were deemed compliant if they received ≥5 palivizumab doses without gaps (>35 days) and their first dose was received by November 30. RSV-related hospitalizations were identified using ICD-9-CM diagnosis codes and examined over 2 observation periods: post-index dose and RSV season. A Cox proportional hazard model was used to evaluate the association between non-compliance and RSV-related hospitalization.

**Results:**

Of the 5,003 subjects who received palivizumab, 62% were deemed non-compliant. Non-compliant subjects had significantly higher unadjusted rates of RSV-related hospitalizations compared to compliant subjects during both observation periods (post-index: 6.1 vs. 2.8 per 100 infant seasons, p < 0.001; RSV season: 5.9% vs. 2.3%; p < 0.001). In multivariate analyses, non-compliance was significantly associated with higher risk of RSV-related hospitalization (HR = 2.01; p < 0.001). Of the 225 RSV-related hospitalizations observed during the RSV season, 61 (27%) occurred before the first dose of palivizumab.

**Conclusions:**

Subjects who did not receive monthly dosing of palivizumab throughout the RSV season had significantly higher rates of RSV-related hospitalizations. The RSV-related hospitalizations prior to the first dose of palivizumab suggest some dosing was started too late.

## Background

Respiratory syncytial virus (RSV) is recognized as the leading cause of serious lower respiratory tract disease in infants and children [1-6]. Conditions associated with a high risk for serious RSV disease include preterm birth (≤35 weeks gestational age [wGA]); chronic lung disease of prematurity (CLDP)/bronchopulmonary dysplasia (BPD]); hemodynamically significant congenital heart disease (CHD); immunodeficiency; and congenital abnormalities of the airways or neuromuscular diseases [7-9].

Palivizumab is a humanized murine monoclonal antibody that is approved by the US Food and Drug Administration (FDA) for the prevention of severe respiratory tract disease caused by RSV in children with BPD, infants with a history of premature birth (≤35 wGA), and children with hemodynamically significant CHD. The FDA-approved dose is 15 milligrams per kilogram administered via intramuscular injection every 28–30 days throughout RSV season. Randomized controlled clinical trials of palivizumab versus placebo have shown a reduction in the incidence of hospitalization due to severe RSV disease by approximately 40%-80% in high-risk premature infants and certain children with CLD or CHD [7,10]. The efficacy of palivizumab at doses <15 mg per kg, or of dosing less frequently than monthly throughout the RSV season, has not been established.

The key objective of this analysis was to examine the association between compliance with palivizumab and risk of RSV-related hospitalization among commercially insured infants.

## Methods

### Data sources

The study used the Optum Research database, which contains de-identified medical and pharmacy claims for a geographically diverse and representative US privately insured population. Enrollees’ claims covered the period from May 01, 2003 through April 30, 2009. All data were accessed using HIPAA [11]-compliant protocols. Because this study did not involve the collection, use, or transmittal of individually identifiable data, Institutional Review Board review or approval was not required.

### Study sample

Commercially insured members with medical and pharmacy benefits who received at least 1 dose of palivizumab (identified with Current Procedural Terminology/Healthcare Common Procedure Coding System codes 90378, C9003, S9562; and National Drug Code identifiers in pharmacy claims) between October and April 2003–2009 were eligible for inclusion. Eligible subjects were in their first year of life with continuous enrollment in the health plan from their date of birth through April 30 of the following year. Inpatient claims do not capture hospital-administered drugs including doses of palivizumab that would be provided at discharge to eligible infants during the RSV season. To ensure the ability to clearly identify all doses, infants born during the RSV season (October 1 – April 30) were excluded from the study. Claims were analyzed from birth through April 30 of the following year.

**Figure 1:**
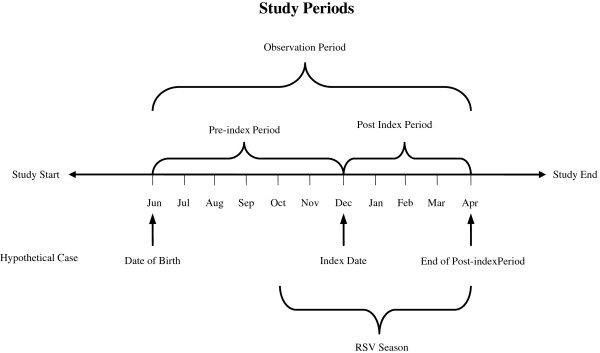
Study periods.

The date of the first palivizumab claim after October 1 was defined as the index date. The pre-index period was defined as the date of birth to the index date. We defined two observation periods for RSV outcomes. The first was the post-index period, defined as the index date through April 30 of the following year. The second was the RSV season, defined as October 1 through April 30 of the following year (Figure 1).

### High-risk populations

High-risk populations were defined as: infants <33 wGA; infants between 33 and 36 wGA; infants with CLD; and infants with hemodynamically significant CHD. Gestational age was defined using International Classification of Diseases, Ninth Revision, Clinical Modification (ICD-9-CM) diagnosis code 765.2x. Subjects with CLD were identified through medical claims with a diagnosis code for CLD and either a pharmacy claim for CLD medications (bronchodilators and/or diuretics), or a claim for oxygen use during the pre-index period. Subjects with CHD were identified through a medical claim with a diagnosis code for CHD and a pharmacy claim for a CHD medication during the pre-index period. High-risk categories were not mutually exclusive groups.

### Outcome measures

The total number of palivizumab doses received during the RSV season was recorded for each subject along with the calendar month of the index dose and the average and maximum number of days between each dose received. Subjects were deemed compliant if they received ≥5 doses of palivizumab with no gaps (>35 days) between doses, and they received their index dose by November 30.

The major outcome of interest was RSV-related hospitalizations, identified using RSV-specific diagnosis codes (079.6, 466.11, 480.1) and RSV-like diagnosis codes defined as unspecified bronchitis (466.0), bronchiolitis (466.19), viral pneumonia (480.9), bronchopneumonia (485), and pneumonia (486) that occurred during the RSV season [12-14]. RSV-like diagnoses were excluded if there were any claims for influenza or other bacterial pneumonia (481, 482.xx, 487.x) within 3 days of the RSV-like claim.

Pre-index clinical characteristics and healthcare utilization were identified to control for potential confounding variables. To account for variable pre-index and post-index periods, all healthcare utilization was calculated per subject per month.

### Univariate analysis

All study variables were analyzed descriptively. Numbers and percentages are provided for categorical variables. Means, medians, and standard deviations are provided for continuous variables. RSV-related hospitalizations for the post-index period were calculated as rates per 100 infant seasons to adjust for the variable length of follow-up time for each individual. Infant seasons were defined as the number of days in the follow-up (end of the season – date of first in-season injection +1) divided by 212 (the number of days in the season). Comparisons were made using t-tests for continuous variables and chi-squared tests for categorical variables.

### Multivariate analysis

Multivariate analysis of RSV-related hospitalizations in the post-index period was conducted using a Cox proportional hazard regression. Parametric regression survival models were based on maximum likelihood estimation. Covariates were entered into the model in variable blocks; if one variable from the block was included, then all variables in the block were included. The first model examined only non-compliance vs. hospitalization. Chronic lung disease, CHD, and gestational age were added to subsequent models. All variables in the final model were tested for collinearity to ensure the accuracy of the parameter estimates. The results of Cox proportional hazard regression are presented as hazard ratios associated with each independent variable.

To understand the stability of the results, the final model was run in population subsets to examine whether the direction or magnitude of the relationship between non-compliance and RSV-related hospitalizations changed in specific population cohorts. The subsets examined were: subjects with CHD, subjects with CLD, subjects whose index dose was received in either October or November, subjects without a pre-index palivizumab dose, subjects categorized by RSV season (e.g., Oct 1, 2003 through April 30, 2004 season), and subjects categorized by birth month.

## Results

### Pre-index clinical characteristics

A total of 5,003 subjects met study inclusion criteria. Over half (57.1%) were single births, with slightly more males (54.7%) than females (Table 1). The subjects’ birth months were evenly distributed across May through August with approximately 21% born in each month except for a slightly smaller proportion (13.1%) born in September.

**Table 1:** Pre-index demographic and clinical characteristics

**Demographic**		**Total**	**Compliant**	**Non-compliant**	**p-value**
		**(N = 5,003)**	**(N = 1,912)**	**(N = 3,091)**	
**Gender**					
Male	n	2,738	1,046	1,692	
	%	54.73	54.71	54.74	0.982
**Birth Number**					
All stillborn siblings	n	25	9	16	
	%	0.5	0.47	0.52	0.819
Single birth	n	2,857	1,049	1,808	
	%	57.11	54.86	58.49	0.012
Twin	n	1,427	599	828	
	%	28.52	31.33	26.79	<0.001
Multiple birth	n	256	115	141	
	%	5.12	6.01	4.56	0.023
Other	n	4	1	3	
	%	0.08	0.05	0.1	0.586
Not available	n	434	139	295	
	%	8.67	7.27	9.54	0.005
**Clinical Characteristics**					
Low birth weight (<2500 g)	n	3,337	1,337	2,000	
	%	66.7	69.93	64.7	<0.001
<33 wGA	n	1,895	755	1,140	
	%	37.8	39.5	36.9	0.065
33-36 wGA	n	2,201	854	1,347	
	%	44	44.7	43.6	0.452
**NICU**					
NICU hospitalization	n	3,830	1,523	2,307	
	%	86.79	88.19	85.89	0.028
NICU hospitalization length	n	4,413	1,727	2,686	
	mean	15.79	15.93	15.7	0.714
	SD	20.38	20.23	20.48	
	median	8	8	8	
**Birth Hospitalization**					
Length of birth hospitalization	n	4,413	1,727	2,686	
	mean	25.4	26.15	24.92	0.062
	SD	21.44	20.98	21.72	
	median	19.00	20.00	18.00	
**Comorbidity**					
Immunodeficiency state	n	23	3	20	
	%	0.46	0.16	0.65	0.013
Trisomy 21	n	119	44	75	
	%	2.38	2.3	2.43	0.778
Intraventricular hemorrhage	n	397	158	239	
	%	7.94	8.26	7.73	0.499
Necrotizing enterocolitis	n	114	35	79	
	%	2.28	1.83	2.56	0.095
Hydrocephalus	n	56	19	37	
	%	1.12	0.99	1.2	0.507
Periventricular leukomalacia	n	54	21	33	
	%	1.08	1.1	1.07	0.919
Retinopathy of prematurity	n	1,206	452	754	
	%	24.11	23.64	24.39	0.545
Sensorineural hearing loss	n	66	20	46	
	%	1.32	1.05	1.49	0.183
Severe neuromuscular disorders	n	127	48	79	
	%	2.54	2.51	2.56	0.921
Cerebral palsy	n	12	3	9	
	%	0.24	0.16	0.29	0.345
Cystic fibrosis	n	60	30	30	
	%	1.2	1.57	0.97	0.059
CMV	n	8	0	8	
	%	0.16	0	0.26	0.026
Chromosomal syndrome	n	168	58	110	
	%	3.36	3.03	3.56	0.316
Congenital anomalies of the airways	n	192	65	127	
	%	3.84	3.4	4.11	0.205
Pre-index home medical equipment	n	1,449	548	901	
	%	28.96	28.66	29.15	0.712
Post-index home medical equipment	n	1,115	430	685	
	%	22.29	22.49	22.16	0.786
Oxygen use	n	392	144	248	
	%	7.84	7.53	8.02	0.529

Approximately two-thirds (66.7%) of the total sample had low birth weight (<2500 g), and a third (36.1%) had at least 1 prespecified comorbidity. The most common comorbidities were retinopathy of prematurity (24.1%), intraventricular hemorrhage (7.9%), and congenital airway anomalies (3.8%) (Table 1).

### Palivizumab utilization

**Figure 2:**
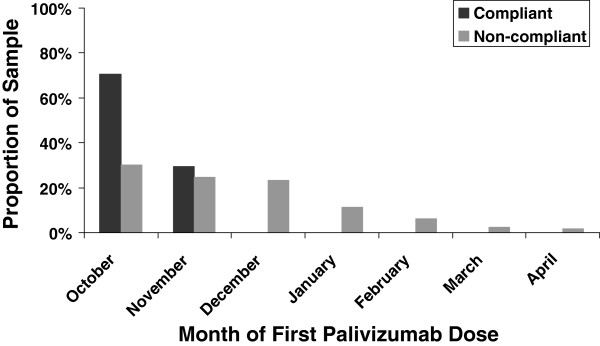
Timing of first palivizumab dose by compliance status.

Overall, 75% of subjects received at least 5 palivizumab doses, and the mean (SD) number of days between doses was 31.6 (8.5). Eighty-seven percent of the index palivizumab doses were given in October (58.7%) or November (28.2%) (Figure 2). On average, subjects had 1.5 dosing gaps, with a mean (SD) gap of 9.4 days (11.4) or 44.4 days since prior dose.

### Clinical characteristics by compliance status

Thirty-eight percent of subjects were deemed compliant. When compared to non-compliant subjects, a higher percentage of compliant subjects were twins (31.3% vs. 26.8%; p ≤ 0.001) and had low birth weight (69.9% vs. 64.7%; p ≤ 0.001). There was no significant difference in the proportion of patients with at least 1 pre-existing comorbidity (p = 0.603). While uncommon, a significantly higher proportion of non-compliant subjects had a diagnosis of immunodeficiency state (0.65% vs. 0.16%; p = 0.013), while a significantly higher proportion of compliant subjects had a diagnosis of cystic fibrosis (1.57% vs. 0.97%; p = 0.059 [Table 1]). Both groups had similar proportions of subjects with retinopathy of prematurity. A significantly higher proportion of compliant subjects had a NICU stay at birth compared to non-compliant subjects (88.2% vs. 85.9%; p = 0.028). There was no statistical difference between compliance groups with regard to time spent in the NICU (p = 0.714) or length of birth hospitalization stays (p = 0.062).

### RSV-related outcomes by compliance status

**Figure 3:**
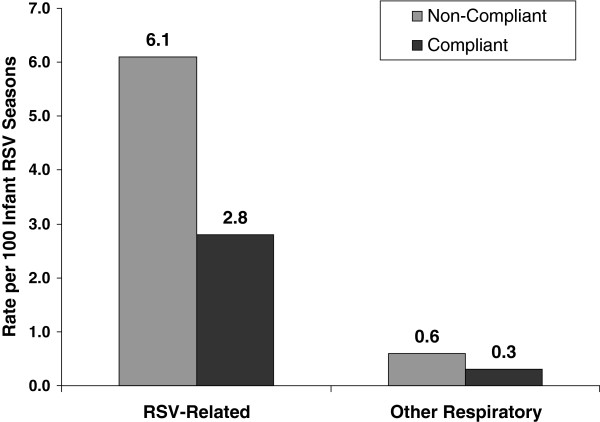
Unadjusted RSV-related hospitalization rate for post-index period by type of hospitalization and compliance status.

Among the total sample (n = 5,003), 164 subjects (3.3%) had at least 1 RSV-related hospitalization during the post-index period. Non-compliant subjects had significantly higher unadjusted rates of RSV-related hospitalizations (6.1 per 100 infant RSV seasons vs. 2.8 per 100 infant RSV seasons; p < 0.001 [Figure 3]). This difference was seen for both RSV and RSV-like hospitalizations (Table 2).

**Table 2:** Proportion of RSV hospitalizations by diagnosis and observation period

		**Total**	**Compliant**	**Non-compliant**	**p-value**
		**(N = 5,003)**	**(N = 1,912)**	**(N = 3,091)**	
**Post-index Period**					
RSV hospitalization	n	57	7	50	
	%	1.14	0.37	1.62	<0.001
RSV-like hospitalization	n	119	33	86	
	%	2.38	1.73	2.78	0.017
**Full RSV Season**					
RSV hospitalization	n	88	9	79	
	%	1.76	0.47	2.56	<0.001
RSV-like hospitalization	n	154	38	116	
	%	3.08	1.99	3.75	<0.001

A total of 225 subjects (4.5%) had at least 1 RSV-related hospitalization during the entire RSV season. Non-compliant subjects also had significantly higher unadjusted rates of RSV-related hospitalizations (5.9% vs. 2.3%; p < 0.001). This difference was seen for both RSV and RSV-like hospitalizations (Table 2). Twenty-seven percent of all RSV-related hospitalizations captured in the study occurred prior to the index dose of palivizumab. The non-compliant group had a larger proportion of hospitalizations prior to the index dose than the compliant group (24.9% vs. 2.2% of RSV-related hospitalizations, respectively). For the main analysis (post-index period), the 61 RSV-related hospitalizations that occurred prior to the first dose of palivizumab were excluded.

There were no significant differences between non-compliant and compliant subjects with regard to other respiratory-related hospitalizations during the post-index period (0.6 vs. 0.3 per 100 infant RSV seasons; p = 0.285) or during the RSV season (0.3% vs. 0.5%; p = 0.134).

### Multivariate analysis

To control for collinearity in the model, a correlation matrix was created to examine the relationship between all of the covariates. There was a weak correlation between low birth weight and unknown gestational age, as well as weak associations between NICU stays and multiple births. No other significant associations were noted; therefore, no covariates were excluded from the model due to collinearity.

After controlling for potential confounders, the Cox proportional hazard model demonstrated that non-compliance was significantly associated with a higher risk of RSV-related hospitalization (HR = 2.01 [95% CI: 1.39 – 2.89]; Table 3). Having CLD, at least 1 prespecified comorbidity during the pre-index period, a pre-index RSV-related hospitalization, and a pre-index ED visit also were significantly associated with a higher risk of RSV-related hospitalization, while being part of a multiple birth and having received a pre-index palivizumab dose were significantly associated with a lower risk of RSV-related hospitalization (Table 2).

**Table 3:** RSV-related hospitalization risk

**Variable**	**Post-index (Cox)**	
	**Hazard ratio (95% CI)**	P-value*
Palivizumab non-compliance (ref. compliance)	**2.007 (1.392 - 2.894)**	**<0.001**
Chronic lung disease	**2.035 (1.262 - 3.281)**	**0.004**
Congenital heart disease	1.24 (0.778 - 1.978)	0.366
33-36 wGA (ref. < 33 wGA)	0.983 (0.634 - 1.522)	0.937
Unknown wGA (ref. < 33 wGA)	**1.966 (1.206 - 3.206)**	**0.007**
Male gender (ref. female)	1.152 (0.841 - 1.578)	0.378
Geographic region – Northeast (ref. South)	0.813 (0.469 - 1.407)	0.459
Geographic region – Midwest (ref. South)	1.226 (0.854 - 1.759)	0.269
Geographic region – West (ref. South)	0.862 (0.526 - 1.414)	0.557
Index year – 2004 (ref. 2003)	1.027 (0.618 - 1.706)	0.919
Index year – 2005 (ref. 2003)	0.893 (0.528 - 1.513)	0.675
Index year – 2006 (ref. 2003)	0.724 (0.415 - 1.262)	0.254
Index year – 2007 (ref. 2003)	0.827 (0.463 - 1.476)	0.520
Index year – 2008 (ref. 2003)	0.922 (0.534 - 1.591)	0.769
Birth number – multiple birth (ref. single birth)	**0.418 (0.267 - 0.655)**	**<0.001**
Birth number – unknown (ref. single birth)	0.656 (0.313 - 1.373)	0.263
Pre-index palivizumab use	**0.507 (0.276 - 0.933)**	**0.029**
NICU hospitalization at birth (ref. no NICU)	1.354 (0.807 - 2.274)	0.251
Unknown NICU hospitalization at birth (ref. no NICU)	0.875 (0.312 - 2.457)	0.800
Pre-index ambulatory visit	0.677 (0.162 - 2.827)	0.593
Pre-index emergency department visit	**1.746 (1.231 - 2.477)**	**0.002**
Pre-index inpatient hospitalization	1.258 (0.387 - 4.085)	0.703
Pre-index RSV-related hospitalization	**3.059 (1.574 - 5.942)**	**0.001**
Any pre-index comorbid condition	**2.003 (1.411 - 2.843)**	**<0.001**
Low birth weight (<2500 g)	0.906 (0.598 - 1.373)	0.643
Birth month: May (ref. Sept)	1.019 (0.587 - 1.769)	0.947
Birth month: June (ref. Sept)	0.7 (0.394 - 1.244)	0.224
Birth month: July (ref. Sept)	0.794 (0.456 - 1.384)	0.416
Birth month: August (ref. Sept)	0.713 (0.405 - 1.256)	0.242

*Values that reflect statistical significance at P<0.05 are in boldface.

*CI*, Confidence interval.

### Sensitivity analyses

**Figure 4:**
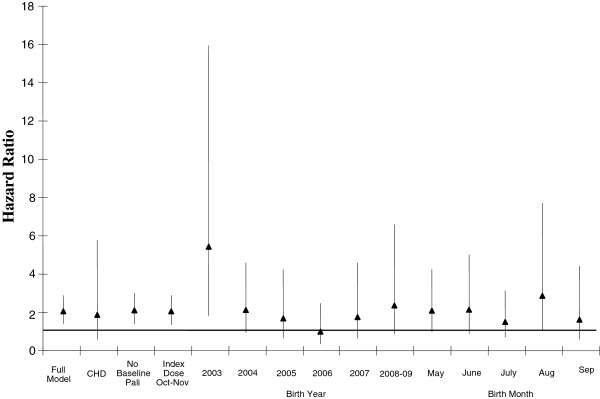
Hazards for RSV-related hospitalization associated with palivizumab non-compliance for select population subsets*. *Data representing CLD have been omitted from this graph due to small sample size and large confidence interval (3.3 – 79.9).

In the majority of sensitivity analyses, there was an association between non-compliance and an increased risk of RSV-related hospitalization although the results were not always statistically significant (Figure 4).

As an additional analysis, we examined a logistic regression model for the full RSV season using the same variables as those used in the Cox proportional hazard model. Using this approach, non-compliance was significantly associated with increased odds of RSV-related hospitalization (OR = 2.45 [95% CI: 1.73 – 3.46]).

## Discussion

The aim of this study was to examine the association between compliance with palivizumab and RSV-related hospitalizations in commercially insured infants. In our study, non-compliant subjects had higher unadjusted rates of RSV-related hospitalizations than compliant infants. The multivariate analysis found that, after adjusting for potential confounding factors, non-compliant subjects were significantly more likely to have an RSV-related hospitalization than compliant subjects. While our analysis cannot determine causality, it identifies an association that should be further explored.

This is one of the largest published studies in a commercially insured population to examine the association between compliance with palivizumab and RSV-related hospitalizations. Previous studies have examined the predictors of compliance with palivizumab, RSV-related hospitalization rates among high-risk infants, and costs and resource use associated with RSV-related hospitalization and/or palivizumab [15-22]. However, we are unaware of other published studies using multivariate analyses to test the association between compliance with palivizumab use and RSV-related hospitalization in a commercially insured population.

In a retrospective claims analysis, Diehl et al. [21] examined the impact of palivizumab compliance on respiratory-related and RSV-specific hospitalizations. Overall, they found 30% of the 245 infants included in their study to be compliant with palivizumab. Unlike the current study, the authors found no significant differences in respiratory-related or RSV-specific hospitalizations between the compliance groups. Their study differs from ours in a number of ways. Their study was an exploratory and descriptive analysis and they did not include multivariate analyses [21]. They analyzed only one season of data with a substantially smaller sample size; and their study sample contained several populations not included in our study such as Medicaid-insured infants, second-season infants, and in-season births, in whom compliance is more difficult to assess.

Despite a large number of subjects receiving 4 or more doses, our study showed significant differences in RSV-related hospitalization rates between compliance groups. On average, subjects had 1.5 dosing gaps (time between doses >35 days) with a mean gap length of 9.4 days or 44.4 days since the previous dose, suggesting that dosing gaps do not need to occur often or be lengthy to be associated with an increased risk of RSV-related hospitalization. Although a relatively small time period, gaps of approximately 15 days would be expected to lead to significantly lower serum levels of palivizumab [23]. Results published by the Palivizumab Outcomes Registry Group [13] demonstrated similar results. The Registry study assessed compliance and RSV-related hospitalization rates in high-risk children receiving palivizumab at home vs. an outpatient setting; the investigators found significantly lower odds of an RSV-related hospitalization in infants receiving all doses within 35 days of previous dose compared with those who did not [16]. A study by Golombek et al. [18] also examined compliance among infants receiving palivizumab at home vs. in-office injections and found that better compliance with home injections was associated with a decrease in the rate of RSV related hospitalizations.

RSV-related hospitalizations prior to the index palivizumab dose occurred in both groups, with significantly more hospitalizations occurring before the index dose in the non-compliant group. The timing of the RSV season varies considerably by region [24], and the presence of early-season hospitalizations underlines the need for effective local monitoring of RSV activity and expediting administration of the first outpatient dose of palivizumab for eligible infants and children. While we selected a palivizumab start date of November 30 to define compliance, it is not possible to determine the actual RSV season start from claims data. Where available, it is best to use local virology and to start dosing infants at high risk of serious RSV disease as early in the season as possible. Likewise, close monitoring should be performed at the end of the season to note when it is appropriate to stop dosing.

### Limitations

Interpretation of our study results must take into account the limitations of the study design and the use of healthcare claims. Administrative claims data are collected for payment purposes and not research; therefore, the degree to which claims accurately describe an individual’s medical history is limited. Claims data also do not contain information on behavioral or social risk factors. Information regarding the subjects’ race, ZIP code level data, and the parents’ socioeconomic status was also unavailable. The association between compliance and RSV-related hospitalization may be confounded by risk factors that we cannot account for in our model.

We relied on diagnosis codes to determine high-risk cohorts. For gestational age, the ICD-9-CM codes are defined in 2-week segments (e.g., 33–34 wGA, 35–36 wGA, etc.). We used a combination of diagnosis codes and medication use to define CLD. CLD is broadly defined as any pulmonary condition resulting from a neonatal respiratory disorder. BPD is a form of CLD, but not all CLD is BPD. CLD is often used interchangeably with BPD. Therefore, some of the identified high-risk cohorts may include infants outside of the labeled indication for palivizumab (e.g., 36 wGA subjects in the 33–36 wGA cohort and non-CLD/BPD subjects in the CLD cohort).

Given hospital coding practices, palivizumab doses administered during a hospitalization were not captured. Therefore, infants born during the RSV season, who are often at highest risk of serious RSV disease, were excluded from the study. Omission of this group may limit the ability to generalize study results to all infants receiving prophylaxis.

RSV is often not tested for during the season; therefore, claims may result in a non-specific ICD-9 diagnosis code. A recent analysis [14] found that only 20% of children with a bronchiolitis episode were tested for RSV, of which half were positive, highlighting the potential underestimation of RSV when solely relying on RSV diagnostic codes. The authors estimated that if the RSV positivity rate among untested bronchiolitis was conservatively half that of infants who were tested, this would result in an underestimation of RSV disease of ~65%. In an attempt to capture all cases, we included RSV-like hospitalizations where it was not possible to confirm an RSV diagnosis. The use of non-specific diagnosis codes may have included hospitalizations unrelated to RSV. We attempted to mitigate potential overestimation by excluding cases where influenza or bacterial pneumonia was diagnosed within 3 days of the RSV-like hospitalization. This is similar to the methodology the US Centers for Disease Control and Prevention uses to estimate RSV burden [25]. While overestimation may have occurred, it is unlikely this bias would impact one cohort more than the other. Since all infants in the analysis were receiving palivizumab, their physicians had identified them as being at high-risk for serious RSV disease and eligible for prophylaxis. Therefore, we do not anticipate the likelihood of testing and/or being given an RSV-specific diagnoses would differ between compliant and non-compliant infants. We also saw consistent trends for an association between increased risk of hospitalization and non-compliance when using both RSV-specific and RSV-related diagnosis codes. It also is assuring that both groups had no significant differences in other respiratory hospitalizations as palivizumab should not affect those hospitalizations.

Our main analysis focused on RSV outcomes that occurred after the first palivizumab dose, defined as the post-index period. In this observation period, there may be differences in RSV exposure time between the compliant and non-compliant cohorts. For example, compliant infants could have the number of days at risk inflated due to adding days early in the season with a lower exposure risk. While non-compliant infants have fewer days at risk, they could have a higher percentage of days in the “peak” RSV period. One of the limitations to using administrative insurance claims is the inability to determine when RSV began circulating in the communities of the subjects included in our analysis. In order to address the potential issues related to the variable exposure/follow-up, we also looked at RSV hospitalizations for a uniform observation period (October 1 to April 30). In both analyses, we saw a statistically significant increased risk/odds of RSV-related hospitalizations associated with non-compliance. Future analyses examining the association between compliance and RSV hospitalization may want to consider collecting data on RSV circulation to adjust for exposure.

In the full RSV season analysis, over a quarter of all RSV-related hospitalizations occurred prior to the first dose of palivizumab. Starting dosing late contributes to non-compliance, so we felt it was important to include hospitalizations that occurred prior to initiation of palivizumab prophylaxis in the analysis. However, hospitalizations prior to the first dose of palivizumab do not tell us the impact of partial prophylaxis on RSV hospitalizations once dosing has been started. For this reason, we chose the post-index observation period as our primary analysis. In the post-index period, we only examined RSV-related hospitalizations that occurred after infants had received their first dose of palivizumab and excluded hospitalizations (n = 61) that occurred prior to the first dose. We did not censor or exclude infants who had an RSV hospitalization prior to the first dose of palivizumab as children can become infected and hospitalized with RSV multiple times in one season. In both the full season and post-index period, we saw a statistically significant increased risk/odds of RSV-related hospitalizations associated with non-compliance.

Finally, our data pertain to a commercially insured population. Therefore, the results are primarily applicable to commercially insured infants and may not be generalizable to other populations such as Medicaid or uninsured infants.

## Conclusions

To our knowledge, this is one of the largest studies in a commercially insured population to examine the association between palivizumab compliance and RSV-related hospitalizations in a clinical setting. In our study, non-compliant subjects had higher unadjusted rates of RSV-related hospitalization than compliant subjects and non-compliance was associated with a significantly higher risk of RSV-related hospitalization after adjusting for potential confounders.

The association between compliance and RSV-related hospitalization and the occurrence of hospitalizations prior to the first palivizumab dose suggest the importance of getting a first dose early in the RSV season and monthly dosing throughout the season. Future analyses should consider other predictors of compliance such as behavioral and social risk factors when examining the association between compliance and RSV-related hospitalization.

## Competing interests

JS, BP, and LB are employees of Optum; KR is an employee of MedImmune and has received stock/stock options from MedImmune and AstraZeneca. DS and MF have served as consultants to MedImmune and have participated as members of MedImmune advisory boards. DS is on the speakers’ bureau for MedImmune. The authors have indicated that they have no other conflict of interest with regard to the content of this article.

## Authors’ contributions

DS and MF collaborated in the design the study, interpretation of results, and drafting and review of the manuscript. KR conceived of the study, collaborated in the design of the study and interpretation of the results, participated in drafting and review of the manuscript. LB developed the programming and conducted the statistical analysis. BP collaborated in the design of the study, directed the analysis, participated in interpretation of the results, and helpt to draft the manuscript. JS participated in drafting of the manuscript. All authors read and approved the final manuscript.

## Pre-publication history

The pre-publication history for this paper can be accessed here:

http://www.biomedcentral.com/1471-2334/13/334/prepub
